# Underwater versus conventional endoscopic mucosal resection for small size non-pedunculated colorectal polyps: a randomized controlled trial (UEMR vs. CEMR for small size non-pedunculated colorectal polyps)

**DOI:** 10.1186/s12876-020-01457-y

**Published:** 2020-09-23

**Authors:** Zhixin Zhang, Yonghong Xia, Hongyao Cui, Xin Yuan, Chunnian Wang, Jiarong Xie, Yarong Tong, Weihong Wang, Lei Xu

**Affiliations:** 1 College of Medicine, Ningbo University, Ningbo, 315211 China; 2 Department of Gastroenterology, Ningbo First Hospital, Ningbo, 315010 China; 3 Department of Gastroenterology, Ninghai Second Hospital, Ningbo, 315600 China; 4 Department of Gastroenterology, Haishu Second Hospital, Ningbo, 315000 China; 5 Ningbo Clinical and Pathological Diagnosis Center, Ningbo, 315021 China

**Keywords:** Underwater endoscopic mucosal resection, Conventional endoscopic mucosal resection, Colonic polyps, Colorectal cancer, Endoscopy

## Abstract

**Background:**

Underwater endoscopic mucosal resection (UEMR) is a recently developed technique and can be performed during water-aided or ordinary colonoscopy for the treatment of colorectal polyps. The objective of this clinical trial was to evaluate the efficacy and safety of UEMR in comparison with conventional endoscopic mucosal resection (CEMR) of small non-pedunculated colorectal polyps.

**Methods:**

Patients with small size, non-pedunculated colorectal polyps (4–9 mm in size) who underwent colonoscopic polypectomy were enrolled in this multicenter randomized controlled clinical trial. The patients were randomly allocated to two groups, an UEMR group and a CEMR group. Efficacy and safety were compared between groups.

**Results:**

In the intention-to-treat (ITT) analysis, the complete resection rate was 83.1% (59/71) in the UEMR group and 87.3% (62/71) in the CEMR group. The en-bloc resection rate was 94.4% (67/71) in the UEMR group and 91.5% (65/71) in the CEMR group (difference 2.9%; 90% CI − 4.2 to 9.9%), showed noninferiority (noninferiority margin − 5.7% < − 4.2%). No significant difference in procedure time (81 s vs. 72 s, *P* = 0.183) was observed. Early bleeding was observed in 1.4% of patients in the CEMR group (1/71) and 1.4% of patients in the UEMR group (1/71). None of the patients in the UEMR group complained of postprocedural bloody stool, whereas two patients in the CEMR group (2/64) reported this adverse event.

**Conclusion:**

Our results indicate that UEMR is safer and just as effective as CEMR in En-bloc resection for the treatment of small colorectal polyps as such, UEMR is recommended as an alternative approach to excising small and non-pedunculated colorectal adenomatous polyps.

**Trial registration:**

Clinical Trials.gov, NCT03833492. Retrospectively registered on February 7, 2019.

## Background

Colorectal cancer (CRC) is among the most common malignancies and remains the second leading cause of cancer-related death globally [1]. It has been well accepted that a majority of CRC cases arise from benign lesions in the colon, mainly colon polyps, and that early removal of colon polyps with endoscopic mucosal resection (EMR) can reduce CRC-related mortality [2]. It has been of note in our daily clinical practice that small size colon polyps are very common. However, conventional endoscopic mucosal resection (CEMR) has a number of limitations. For example, CEMR usually requires submucosal injection, which may displace the polyp into a less accessible location or constrict the lumen, making it more difficult to access the lesion, and there is the risk of dysplastic seeding into deeper wall layers [3].

Despite an improvement in the removal of small size colon polyps with following the application of cold snare polypectomy (CSP) [4] or cold biopsy forceps (CBF) [5], the complete resection rate varies significantly among different studies. Recently, a new technique of water-immersion EMR, referred to as underwater endoscopic mucosal resection (UEMR), was described by Binmoeller et al. [6] The procedure can be performed under water-aided or ordinary colonoscopy for the treatment of colorectal polyps. UEMR replaces inflation with the use of water to fill the intestinal cavity, thus avoiding submucosal injection and carrying the lesion away from the submucosal layer. In recent years, numerous studies have assessed the safety and efficacy of this technique for the resection of large colorectal lesions [7–12]. The results obtained suggest that UEMR is effective and safe for treating large and medium-size colorectal polyps [7–9]. In addition, UEMR has been reported as a new approach for the removal of colorectal adenomas of the appendiceal orifice. Compared with CEMR, UEMR is associated with a higher complete excision rate and lower risk of developing procedure-related adverse events [9], and it is applied for small colorectal adenomas even in Japan [13]. Despite the advantages, questions remain as to whether UEMR can be used as an alternative to CEMR in the treatment of small colorectal polyps. To date, there has been no prospective randomized controlled clinical trial to focus on evaluate its efficacy and safety in the resection of small size colorectal polyps.

In this randomized controlled clinical trial, we aimed to compare CEMR with UEMR in terms of safety and effectiveness in the removal of small size, non-pedunculated colonic polyps.

## Methods

### Patients and study design

The study is multicentric, parallel-group, open-label, randomized, non-inferiority comparative trial, which was designed according to the Consolidated Standards of Reporting Trials (CONSORT) guideline [14] (See Supplementary Table 1, Additional File 1). In this clinical trial, the patients were recruited from three hospitals: (A) Ningbo First Hospital, Ningbo, China (Institution A); (B) Ninghai Second Hospital, Ningbo, China (Institution B); and (C) Haishu Second Hospital, Ningbo, China (Institution C). During enrollment, all consecutive outpatients 18–75 yr of age who had undergone colonoscopy and had at least one non-pedunculated colorectal polyp (4–9 mm in size) were included in the study. Patients with the following conditions were excluded from the clinical trial: (1) pregnancy; (2) inflammatory bowel disease (IBD); (3) familial polyposis; (4) severe organ failure; (5) taking anticoagulant or antiplatelet medications; (6) unwillingness to provide written informed consent; (7) colorectal polyps with clinical signs of a deep submucosal invasion. Patients with colorectal polyps ≥10 mm referred for routine treatment.

This clinical study was conducted in accordance with the guidelines of the Declaration of Helsinki. Written informed consent had been obtained from all participants prior to inclusion in the study. The study protocol was reviewed and approved by the medical ethics committee of Ningbo first hospital (2019-R008). In addition, approval has been obtained from all of the participating hospitals: Ninghai Second Hospital and Haishu Second Hospital. The study was registered with Clinical Trials.gov (NCT 03833492).

### Treatment and follow-up

Simple randomization strategy was performed, and the study patients were randomly assigned at a 1:1 ratio to two treatment groups using a Stata-generated (version 13.0, StataCorp LP, College Station, TX) randomized sequence with a serial number assigned to an opaque, sequentially numbered envelope by a staff member otherwise unaffiliated with the study. When a polyp was found during colonoscopy, the assistant opened the randomized envelope to determine which technique should be used for removal. The operating endoscopists were not blinded during the procedures and performed all endoscopic polypectomies using the same model of high-definition video colonoscope (Olympus PCF 290 video colonoscope, Olympus Inc., Japan) and round snare (JHY-SD-23-230-30-A1) in all cases. Prior to endoscopic resection, each study patient received standardized instructions for bowel preparation. None of the patients were anesthetized.

**Fig. 1:**
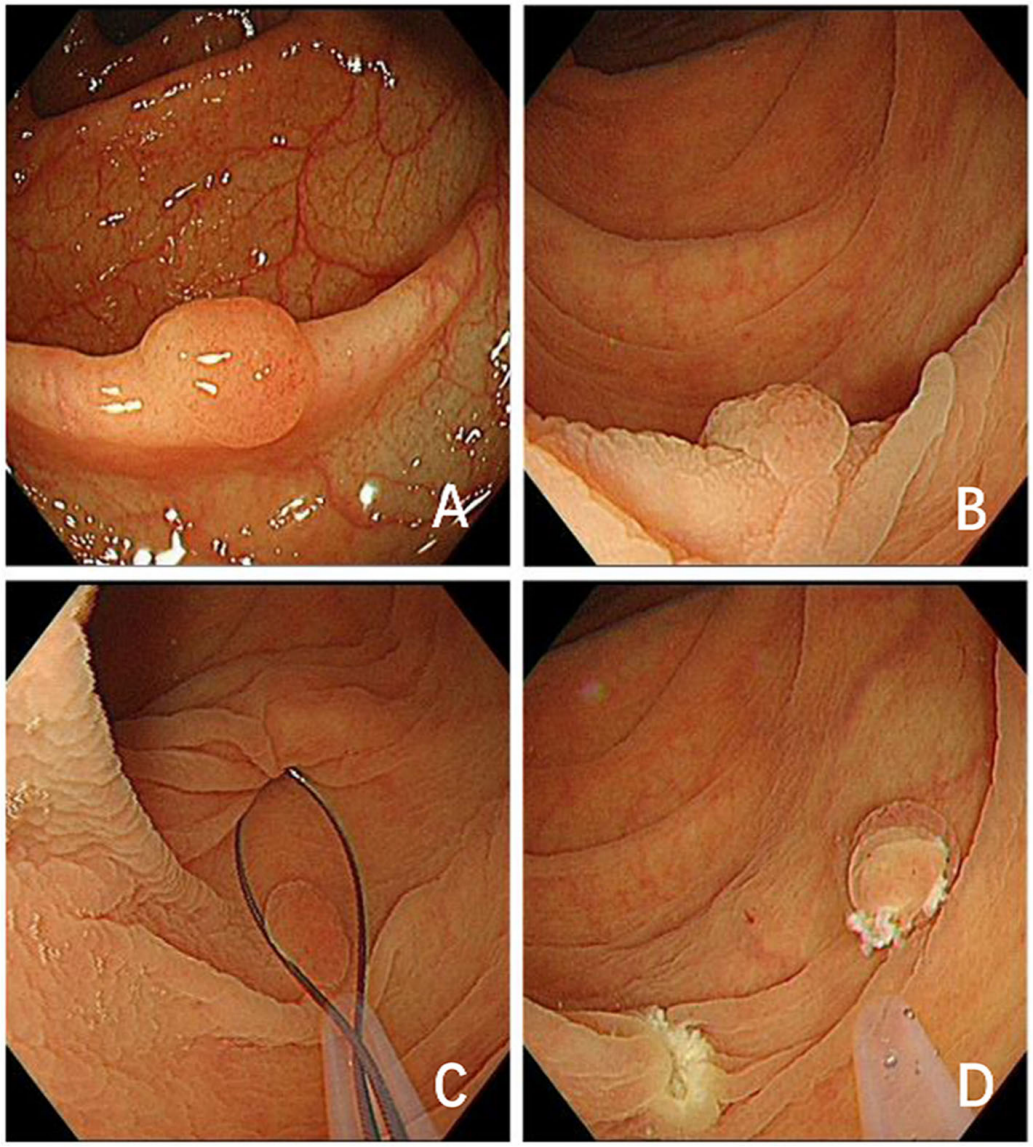
Underwater endoscopic mucosal resection (UEMR). **a** A flat elevated polyp was detected in the colon; **b** The colorectal lumen was completed deflated with sterile water; **c** The polypectomy snare was used for UEMR; **d** Biopsies were obtained from two marginal sites located symmetrically to the left and right of mucosal defects to confirm residual polyp tissue

The process of endoscopic resection was initiated with mucosal inspection during the withdrawal period. Once a target polyp was identified, its characteristics, including location, size, and morphology (based on the Paris classification) [15], were carefully recorded. The CEMR protocol included the following steps, as described in our previous study [16]: (1) injection of normal saline solution into the submucosa; (2) removal of the polyps with an open snare. The UEMR protocol included the following steps: (1) the colorectal lumen was completed deflated with 500–1000 mL sterile water using a flushing pump (Olympus OFP2); (2) the lesion and 2–3 mm of normal mucosa surrounding the base of the polyp were snared and subsequently resected with an electrosurgical generator (VIO200D; ERBE Elektromedizin GmbH, Tübingen, Germany) in Endo cut Q mode: effect 4, interval 6, length 1, and forced coagulation current (output limit 40 W, effect 2) (Fig. 1).

During the endoscopic procedures, the operating endoscopists tried initially to achieve en-bloc resection of the polyps. Otherwise, piecemeal resection was performed until the site was considered devoid of polypoid tissue. Following polyp resection, the edge of the wound was rinsed with water and carefully inspected for residual lesions. If remnant colorectal polyps were suspected or present, residual lesions were resected using the same methodology. If there were no remaining polyps, two cold forceps biopsies were obtained from the margins of the wounds for examination, all polyps need marginal biopsies. Polyps and biopsy specimens were collected and placed separately in formalin containers for subsequent histopathological analysis at each participating hospital.

### Outcome measurements

The primary outcomes were measurements of complete resection rate and en-bloc resection rate. Secondary outcomes included examinations of resection method, duration of the resection, and adverse events (e.g. bleeding, perforation). Resection duration was defined as the period between the start of submucosal injection (in the CEMR group) or intra-intestinal water injection (in the UEMR group) and completion of the colonoscopic resection. Intraprocedural bleeding was defined as any early attack (during the examination) that required endoscopic hemostasis. Endoscopic hemostasis was performed when the active hemorrhage lasted for ≥30 s, regardless of the surgical method (including the coagulation of vessels in the ulcer or clipping of a bleeding postpolypectomy mucosal defect). Resected wounds were closed with clips for preventive hemostasis according to the operator’s preference. Two types of post-polypectomy bleeding (PPB) were evaluated: early PPB (persistent bleeding lasting ≥30 s immediately after polypectomy) and delayed PPB (presence of bloody stool and endoscopic hemostasis during 7–14 days’ follow-up). All patients were followed by telephone within 7–14 day to assess the frequency of adverse postoperative events.

### Statistical analysis

Statistical analysis was carried out using SPSS version 21 (SPSS, Chicago, IL) and Stata version 13.0 (StataCorp LP, College Station, TX). Sample size was calculated on the basis of a previous study. The results obtained for the Chinese patient population included in this study indicated that incomplete resection rate was 1.5% for mucosal or submucosal adenomatous polyps (6–9 mm in size) treated with CEMR [17]. By comparison, an incomplete resection rate of 7.2% has been reported for the treatment of similar polyps with CEMR in one study of a Korean patient population [18]. We estimated incomplete resection rate of 1.5% for both groups in the present study. The non-inferiority margins for comparative analysis between the CEMR and UEMR groups were defined with an absolute risk difference of 5.7%, in order to ensure that the residual rate for the UEMR group would not exceed 7.2%. The following parameters were used to calculate sample size: an α-error level of 0.05 (one-sided), a β-error level of 0.20, and a total of 114 polyps (57 polyps per group). We assumed that approximately 10% of colorectal polyps could be excluded from the analysis set, and the total size of the sample was determined as 130 polyps. In this clinical trial, we enrolled a total of 130 patients with 142 small colorectal polyps.

The primary outcomes of complete resection rate and en-bloc resection rate were determined using both intention-to-treat (ITT) and per-protocol (PP) analyses. As for the complete resection rate and En-bloc resection rate, if the lower bound of the 90% confidence interval (CI) of the risk difference more than − 5.7%, noninferiority of UEMR vs. CEMR group could be concluded. Secondary outcomes were evaluated with ITT analysis. Categorical variables are expressed as percentages (90% confidence interval). Continuous variables are presented as mean ± standard deviation (SD) or median [interquartile range (IQR)], as appropriate. The χ^2^ test was conducted to evaluate the associations between categoric variables. Continuous data were assessed using the t-test. The Wilcoxon rank-sum test was carried out to evaluate procedure time and polyp size. A *p*-value < 0.05 was considered statistically significant.

## Results

### Baseline demographic and clinical characteristics

**Fig. 2:**
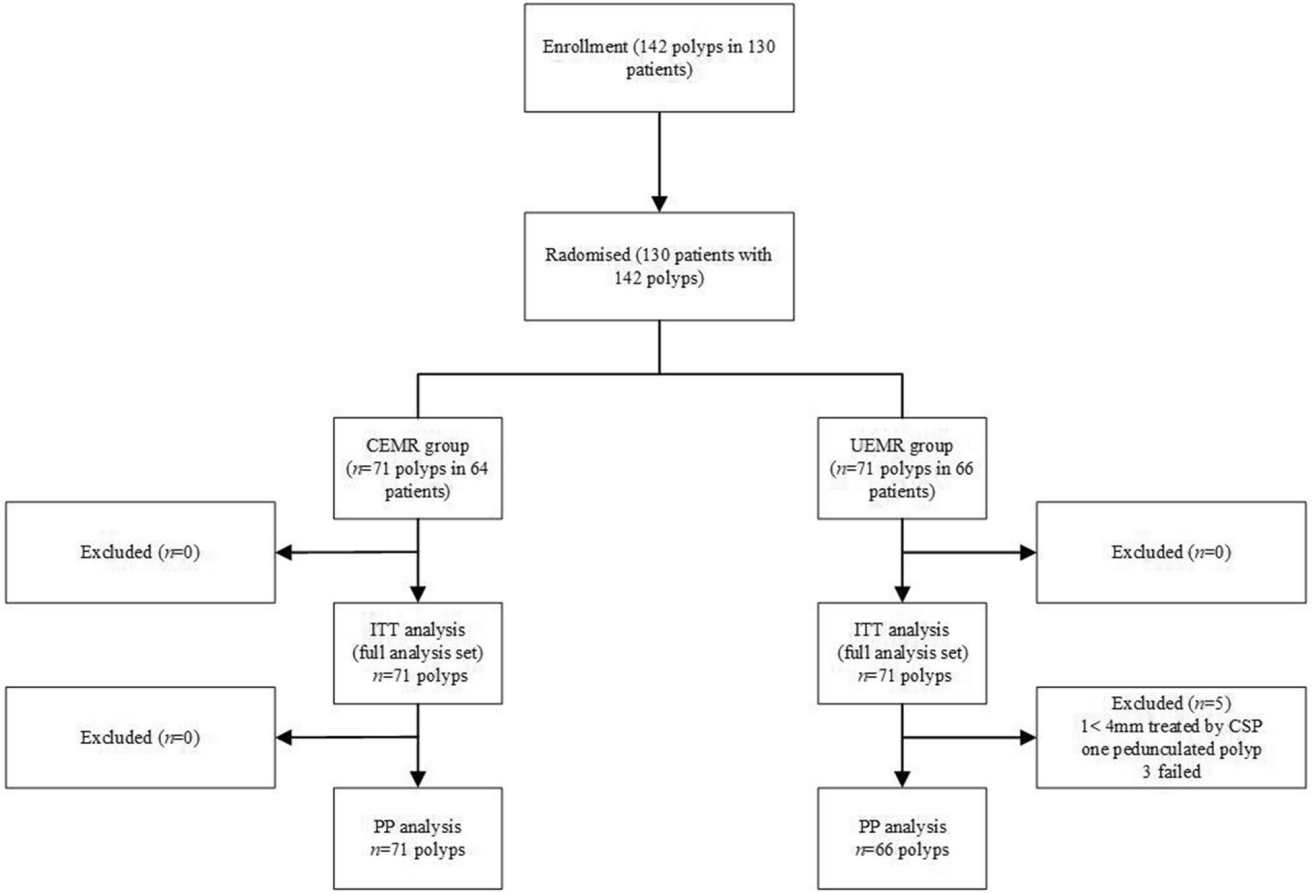
Schematic diagram of patient enrollment and study design. A total of 130 patients with 142 colorectal polyps were allocated randomly to the CEMR or UEMR group

A total of 130 patients and 142 small colorectal polyps (4–9 mm) were enrolled in this clinical trial between May 2019 and November 2019. The baseline demographic and clinical characteristics of the CEMR group (*n* = 64) and UEMR group (*n* = 66) are summarized in Tables 1 and 2. This study had competitive enrollment, and 142 colorectal lesions (71 per group) were eventually included in the ITT analysis of primary outcomes. In the PP analysis, five polyps (one < 4.0 mm polyp, one pedunculated polyp, and three polyps which surgery failed to remove) were excluded from the UEMR group; 125 patients with 137 colorectal lesions (66 in the UEMR group and 71 in the CEMR group) were studied (Fig. 2). The mean age of the study patients was 56.4 yr (SD, 10.6 yr), and 57.7% were male. In general, no significant differences in demographic characteristics were observed between the two groups (Table 1). Altogether, three expert operators participated in this study. The operators’ detailed experience is shown in Table S2.

**Table 1:** Baseline characteristics of the study participants

Parameter	CEMR (*n* = 64)	UEMR (*n* = 66)	Total (*n* = 130)	*P* value
Age, mean (SD), yrs	57.6 ± 9.8	55.1 ± 11.2	56.4 ± 10.6	0.177^a^
Sex, *n* (%)				
Male	35 (54.7)	40 (60.6)	75 (57.7)	0.495^b^
Female	29 (45.3)	26 (39.4)	55 (42.3)	
Current alcohol, *n* (%)				0.866^b^
Yes	13 (20.3)	11 (16.7)	24 (18.5)	
No	39 (60.9)	42 (65.6)	81 (62.3)	
Unknow	12 (18.8)	13 (19.7)	25 (19.2)	
Current tobacco, *n* (%)				0.910^b^
Yes	11 (17.2)	13 (19.7)	24 (18.5)	
No	41 (64.1)	40 (60.6)	81 (62.3)	
Unknow	12 (18.8)	13 (19.7)	25 (19.2)	
Institution, *n* (%)				0.441^b^
A	51 (79.7)	51 (77.3)	102 (78.5)	
B	10 (15.6)	14 (21.2)	24 (18.4)	
C	3 (4.7)	1 (1.5)	4 (3.1)	

*BMI* body mass index, *CEMR* conventional endoscopic mucosal resection, *UEMR*, underwater endoscopic mucosal resection. ^a^ Two-sample t test. ^b^ Chi-square test

Institution A: Ningbo First Hospital, Ningbo, China

Institution B: Ninghai Second Hospital, Ningbo, China

Institution C: Haishu Second Hospital, Ningbo, China

**Table 2:** Colorectal polyps in the study participants

Parameter	CEMR (*n* = 71)	UEMR (*n* = 71)	Total (*n* = 142)	*P* value
Median Size, (IQR, mm)	5.0 (4.0–7.0)	6.0 (5.0–8.0)	6.0 (4.0–7.0)	0.061^a^
Location, *n* (%)				0.612^b^
Ascending colon	16 (22.5)	13 (18.3)	28 (20.4)	
Transverse colon	17 (23.9)	21 (29.6)	38 (26.8)	
Descending colon	5 (7.1)	6 (8.4)	11 (7.7)	
Sigmoid colon	28 (39.4)	22 (31.0)	50 (35.2)	
Rectum	5 (7.1)	9 (12.7)	14 (9.9)	
Morphology, *n* (%)				0.339^b^
0-Is	50 (70.4)	54 (76.1)	104 (73.2)	
0-Ip	0 (0.0)	1 (1.4)	1 (0.7)	
0-IIa	21 (29.6)	16 (22.5)	37 (26.1)	
Neoplastic polyps, *n* (%)				0.618^b^
Tubular	47 (66.2)	48 (67.6)	95 (66.9)	
Tubulovillous or villous	0 (0.0)	2 (2.8)	2 (1.4)	
SSA	1 (1.4)	1 (1.4)	2 (1.4)	
Other polyps, *n* (%)				
Hyperplastic polyps	13 (18.3)	9 (12.7)	22 (15.5)	
Inflammatory polyps	10 (14.1)	11 (15.5)	21 (14.8)	

*CEMR* conventional endoscopic mucosal resection, *UEMR* underwater endoscopic mucosal resection, *SSA* sessile serrated adenoma

^a^ Wilcoxon rank-sum test. ^b^ Chi-square test

The baseline clinical characteristics of the colorectal polyps in the study patients are shown in Table 2. Overall median polyp size was 6.0 mm (range, 4.0–7.0 mm); median in the CEMR group was 5.0 mm (range, 4.0–7.0 mm); median polyp size in the UEMR group was 6.0 mm (range, 5.0–8.0 mm). A large proportion of the colorectal polyps were tubular adenomas (66.9%), of which 20.4% were located on the left of the colon. With regard to morphological features, 73.2% of the colorectal polyps were classified as Is, while the remaining 26.1% were IIa types. Overall, no significant difference in size, gross type, anatomical location, morphological features, or pathologic diagnosis was observed between the two groups (Table 2).

### Primary outcomes after UEMR vs. CEMR

**Fig. 3:**
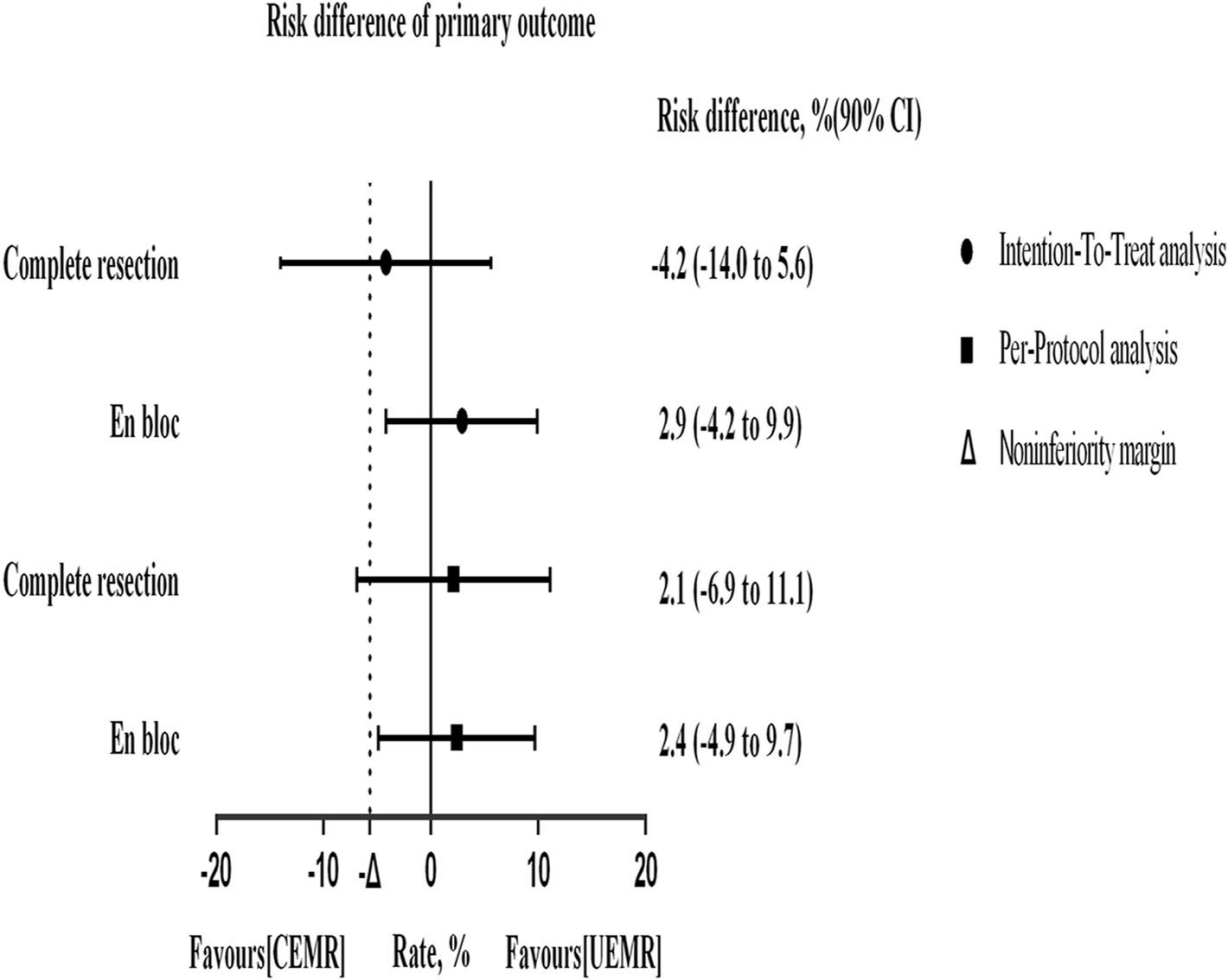
Non-inferiority graph for primary outcomes. Datapoints are the point estimate of the risk difference between the CEMR and UEMR, error bars are 90% CI

We compared the complete resection rate and the en-bloc resection rate of the UEMR group to those of the CEMR group using ITT and PP analysis methods. Complete resection was defined as a complete en-bloc resection of a lesion with tumor-free lateral margins (negative biopsy results from specimens obtained from the resection margin after polypectomy), incomplete resection was defined as at least one neoplastic tissue retrieved from the resection edge after polypectomy. ITT analysis showed that the overall complete resection rates were 87.3% (62/71) in the CEMR group and 83.1% (59/71) in the UEMR group (Table 3), and that the en bloc rates were 91.5% (65/71) in the CEMR group and 94.4% (67/71) in the UEMR group (difference 2.9%; 90% CI − 4.2 to 9.9%), showing noninferiority (noninferiority margin − 5.7% < − 4.2%) of CEMR compared with UEMR (Fig. 3). PP analysis was performed to compare the complete resection rate and the en-bloc resection rate between the UEMR group and the CEMR group (Table 3). The results of ITT and PP analyses indicated the non-inferiority of the UEMR group, compared to the CEMR group in En-bloc resection.

**Table 3:** Comparative analysis of primary outcomes

Parameter	CEMR	UEMR	*P* value
Intention-to-treat analysis	*n* = 71	*n* = 71	
Complete resection, *n*	62	59	
Rate (%) [90% CI]	87.3 [80.7–94.0]	83.1 [75.6–90.6]	0.478^a^
Incomplete resection, *n* (%)	9 (12.7)	12 (16.9)	
En-bloc, *n*	65	67	
Rate (%) [90% CI]	91.5 [86.0–97.1]	94.4 [89.8–99.0]	0.512^a^
Piecemeal, *n* (%)	6 (8.5)	4 (5.6)	
Per-protocol analysis	*n* = 71	*n* = 66	
Complete resection, *n*	62	59	
Rate (%) [90% CI]	87.3 [80.7–94.0]	89.4 [83.0–95.8]	0.706^a^
Incomplete resection, *n* (%)	9 (12.7)	7 (10.6)	
En-bloc, *n*	65	62	
Rate (%) [90% CI]	91.5 [86.0–97.1]	93.9 [89.0–98.9]	0.591 ^a^
Piecemeal, *n* (%)	6 (8.5)	4 (6.1)	

*CEMR* conventional endoscopic mucosal resection, *UEMR* underwater endoscopic mucosal resection

^a^Chi-square test

### Secondary outcomes after CEMR vs. UEMR

Upon comparison of the frequency of adverse events between the UEMR group and the CEMR group, we found that early bleeding was observed in 1.4% (1/71) and 1.4% (1/71) of the CEMR and UEMR groups, respectively. After the procedures, two of the patients in the CEMR group reported bloody stool, whereas no patients complained of this condition in the UEMR group. Furthermore, neither clinically significant post-procedural perforation nor intraprocedural perforation occurred in either group. Overall, the incidence of each adverse event did not differ significantly between the groups.

As shown in Table 4, the median operation time was 81 s (range, 52–113 s) in the CEMR group and 72 s (range, 53–83 s) in the UEMR group. There was no significant difference in duration between the two groups.

**Table 4:** Comparative analysis of secondary outcomes

Parameter	CEMR	UEMR	Total	*P* value
	*n* = 71	*n* = 71	*n* = 142	
Median procedure time (IQR, second)	81 (52–113)	72 (53–83)		0.183^a^
Adverse event	*n* = 71	*n* = 71	*n* = 142	
Bleeding	1	1	2	1.000^b^
Perforation	0	0	0	/
	*n* = 64	*n* = 66	*n* = 130	
Delayed bleeding	2	0	2	0.240^b^
Delayed perforation	0	0	0	/
Unfollowed	4	3	7	0.716^b^

*CEMR* conventional endoscopic mucosal resection, *UEMR* underwater endoscopic mucosal resection

Bleeding defined as prolonged post-polypectomy bleeding (> 30 s)

Delayed bleeding was defined as the presence of bloody stool and endoscopic hemostasis during 7–14 days’ follow-up

^a^Wilcoxon rank-sum test

^b^Two-tailed Fisher exact test

## Discussion

The effectiveness and safety of UEMR in the resection of small size colorectal polyps have not yet been assessed. To our knowledge, this multicenter randomized controlled clinical trial is the first study to compare the effectiveness and adverse effects between CEMR and UEMR in the treatment of small colorectal polyps in a Chinese population. The major novel findings are summarized as follows: (1) the complete resection rate and the en-bloc resection rate of small size colorectal adenomatous polyps (4–9 mm in size) in the UEMR group were similar to those in the CEMR group, without significant differences between the two groups; (2) UEMR was safer, as evidenced by a lower incidence of complications and a flat delayed bleeding rate within 2 wk. following the procedures; (3) These results suggest that UEMR was equally effective and safer compared with CEMR, and thus it holds promise as one of the standard techniques to be used for coloscopic resection of 4–9 mm non-pedunculated colorectal polyps in clinical practice.

In this clinical study, the efficacy of CEMR and UEMR in the treatment of small, non-pedunculated colorectal polyps was evaluated on the basis of residual polyp tissue. Although the definition of the complete resection rate and incomplete resection rate has not been standardized, several previous studies have shown that marginal biopsy was sufficient for small lesions [19, 20]. Therefore, we adopted this method as previously validated, using a biopsy with a resected edge for the examination of residual polyp tissue in the present multicenter clinical trial.

The rate of en-bloc has been shown to be related to the residual rate. Several previous studies suggested that high rates of piecemeal resections led to high recurrence rates [21–23]. In fact, UEMR showed a higher en-bloc resection rate for large colorectal polyps in these previous studies. For colorectal polyps with median size of 12 mm, Kim and colleagues found that the application of UEMR resulted in an en-bloc resection rate of 88.9% [24], which was significantly greater than that of CEMR. For colorectal polyps with an average size of 20.78 mm, Rodríguez Sánchez et al. found that the en bloc resection rate was 62% [8] using UEMR. Although there was no statistical difference, this was significantly higher than the 49.1% observed for CEMR. Notably, we showed that UEMR in the resection of small size polyps had an en-bloc resection rate of as high as 94.4%. Furthermore, it was noted that the en-bloc resection rate of UEMR was not inferior to that of CEMR, regardless of the size of colorectal polyps.

For the coloscopic resection of colorectal polyps 10–20 mm in size, Yamashina et al. [7] showed that the complete resection rate in the UEMR group was greater than that of the CEMR group. For colorectal polyps with median size of 20 mm, studies by Uedo et al. [25] found that the complete resection rate was 64%. For small size polyps of 4–9 mm in size, our study showed that the complete resection rate was as high as 83.1%. But, our results not show noninferiority of UEMR compared with CEMR in complete resection, probably because of the small sample size. Considering the limitations for CEMR, previous efforts have been made, including the application of CSP, hot snare polypectomy (HSP), and other technologies to overcome the shortcomings of CEMR [17, 26]. Our results, together with those of others, have suggested that UEMR, as a newly developed technology, is an emerging method with enormous for the removal of colorectal polyps. However, futher data from large RCTs are needed to prove it effective in complete resection.

It may also merit attention in this clinical trial that no perforation as procedural complication occurred in any case included in the study. Since 2012, only one patient undergoing UEMR developed perforations, as described previously [27]. However, in a report on CEMR, the perforation rate ranged from 1.2 to 4.4% in patients who underwent CEMR [28, 29]. These previous findings further confirm the safety of UEMR. The considerably low incidence of perforation in patients with UEMR may offer evidence in support of the use of UEMR in the treatment of colorectal polyps.

In this study, postoperative bleeding was observed in one patient in the CEMR group and one patient in the UEMR group. In addition, delayed hemorrhage occurred in two patients in the CEMR group, with no patient with this clinical complication in the UEMR group. Our findings were consistent with three previous studies: one reported a delayed bleeding rate of 5% [11], one described a rate of 6.7% [12], and one of 0% [30].

The operative duration in the UEMR group was not significantly different from that of the CEMR group. This result is in disagreement with a previous study [31]. A possible explanation is that the flow rate of the pump used in our study was relatively small, and the time required for the liquid to fill the intestine was similar to that of submucosal injection. This does not happen when we are performing a water-aided colonoscopy because the lumen has already full of water. Thus, this technique seems to be more suitable for water-aided colonoscopy.

In recent years, water-aided colonoscopy has been proven to improve intestinal cleanliness in a number of previous studies [32, 33], and it can improve the detection rate of colorectal adenoma [34]. As for water-aided colonoscopy, UEMR seems to be more convenient than CEMR, the overall operation time can be reduced. Compared to the CEMR, UEMR does not require injection needles, generated greater economic benefits, and be more friendly to patients with poor financial conditions. Our research also found that for patients with poor bowel preparation, UEMR can play a clean role. Although there is no difference between the two groups in terms of the main outcome, it is more appropriate to use UEMR in the above cases.

Our study may have a number of limitations. First, this study was an open multicenter study, but the sample size was relatively small, and the operating endoscopists could not achieve the blind method. In the future, a multicenter randomized controlled study with larger sample size is needed to further verify the results in the present study. Second, three colorectal polyps were not removed with UEMR, mainly due to the location, where they were not well exposed after immersion in water. After attempts were made, CEMR was eventually used for the treatment of these colorectal polyps. It has to be pointed out that all of these failed cases were excluded from the PP analysis. Third, the overall operation time was not compared between the two groups, and further study will be needed to assess whether UEMR could require shorter time than CEMR.

## Conclusions

In conclusion, our findings have demonstrated that UEMR is equally effective in En-bloc resection and safer compared to CEMR for the treatment of small size colon polyps. The scientific evidence is in support of a recommendation for UEMR as an alternative technique for excising small (4–9 mm) non-pedunculated colorectal polyps.

## Supplementary information

**Additional file 1: Table S1.** CONSORT 2010 Checklist.

**Additional file 2: Table S2.** Operators’ experiences.

## Data Availability

The datasets used and/or analysed during the current study are available from the corresponding author on reasonable request.

## Abbreviations

BMI: Body mass index
CBF: Cold biopsy forceps
CEMR: Conventional endoscopic mucosal resection
CI: confidence interval
CRC: Colorectal cancer
CSP: Cold snare polypectomy
EMR: Endoscopic mucosal resection
HSP: Hot snare polypectomy
IBD: Inflammatory bowel disease
IQR: Interquartile range
ITT: In the intention-to-treat
PP: Per-protocol
PPB: Post-polypectomy bleeding
SD: Standard deviation
UEMR: Underwater endoscopic mucosal resection
