# Supplementary material for: Underwater versus conventional endoscopic mucosal resection for small size non-pedunculated colorectal polyps: a randomized controlled trial: (UEMR vs. CEMR for small size non-pedunculated colorectal polyps)
Source: BMC Gastroenterol. 2020 Sep 23;20:311. doi: 10.1186/s12876-020-01457-y (PMC7510164; doi:10.1186/s12876-020-01457-y)
Supplement: Supplementary file 2 — Additional file 2: Table S2. Operators’ experiences. [file 12876_2020_1457_MOESM2_ESM.docx]

**Table S2.** Operators’ experiences

| Operator | Approximate numbers of colonoscopy experienced | Approximate numbers of colonic CEMR experienced | Approximate numbers of colonic ESD experienced |
| --- | --- | --- | --- |
| Yonghong Xia | ≥1000 | ≥300 | <30 |
| Hongyao Cui | ≥1000 | ≥300 | <30 |
| Lei Xu | ≥3000 | ≥1000 | ≥50 |

CEMR, conventional endoscopic mucosal resection; ESD, endoscopic submucosal dissection.
